# Peer Review for " Postacute Sequelae of COVID-19 and Adverse Psychiatric Outcomes: Protocol for an Etiology and Risk Systematic Review"

**DOI:** 10.2196/45308

**Published:** 2023-03-14

**Authors:** Daniel Griffin

**Affiliations:** 1 Columbia University New York City, NY United States

**Keywords:** COVID-19, long COVID, post–COVID-19 condition, postacute sequelae of COVID-19, PASC, psychiatry, epidemiology, evidence-based medicine

*This is a peer-review report submitted for the paper “Postacute Sequelae of COVID-19 and Adverse Psychiatric Outcomes: Protocol for an Etiology and Risk Systematic Review.”*

## Round 2 Review

### General Comments

The authors lay out a reasonable protocol for this type of investigation [1].

### Serious Concerns

*Do you have any serious concerns about the manuscript such as fraud, plagiarism, unethical or unsafe practices?*

No.

*Have authors’ provided the necessary ethics approval (from authors’ institution or an ethics committee)?*

Yes.

### Language Quality

*How would you rate the English language quality?*

High quality.

### Validity and Reproducibility

*Is the reasons for conducting the study and its objectives clearly explained?*

Yes.

*Is the study design appropriate?*

Yes.

*Are sufficient details provided so that the method can be replicated?*

Yes.

*Are datasets available so that others could use them?*

Not applicable.

### Suggestions

*Based on your answers in section 3 how could the author improve the protocol?*

It is appropriate as it is.

*Do you have any other feedback or comments for the Author?*

The authors lay out a reasonable protocol for this type of investigation that is based on a fairly standard approach with the standard GRADE grading approach.

### Decision

*Verified manuscript:* The content is scientifically sound, and only minor amendments (if any) are suggested.
